# Emerging Cancer Vaccines: The Promise of Genetic Vectors

**DOI:** 10.3390/cancers3033687

**Published:** 2011-09-22

**Authors:** Luigi Aurisicchio, Gennaro Ciliberto

**Affiliations:** 1 Takis, via di Castel Romano 100, 00128 Rome, Italy; E-Mail: ciliberto@unicz.it; 2 BIOGEM scarl, via Camporeale, 83031 Ariano Irpino (AV), Italy; 3 Dipartimento di Medicina Sperimentale e Clinica, Università degli studi di Catanzaro “Magna Graecia”, 88100 Catanzaro, Italy

**Keywords:** cancer vaccines, tumor associated antigens, adenoviral vectors, DNA electroporation

## Abstract

Therapeutic vaccination against cancer is an important approach which, when combined with other therapies, can improve long-term control of cancer. In fact, the induction of adaptive immune responses against Tumor Associated Antigens (TAAs) as well as innate immunity are important factors for tumor stabilization/eradication. A variety of immunization technologies have been explored in last decades and are currently under active evaluation, such as cell-based, protein, peptide and heat-shock protein-based cancer vaccines. Genetic vaccines are emerging as promising methodologies to elicit immune responses against a wide variety of antigens, including TAAs. Amongst these, Adenovirus (Ad)-based vectors show excellent immunogenicity profile and have achieved immunological proof of concept in humans. *In vivo* electroporation of plasmid DNA (DNA-EP) is also a desirable vaccine technology for cancer vaccines, as it is repeatable several times, a parameter required for the long-term maintenance of anti-tumor immunity. Recent findings show that combinations of different modalities of immunization (*heterologous* prime/boost) are able to induce superior immune reactions as compared to single-modality vaccines. In this review, we will discuss the challenges and requirements of emerging cancer vaccines, particularly focusing on the genetic cancer vaccines currently under active development and the promise shown by Ad and DNA-EP heterologous prime-boost.

## 1. Introduction

In spite of significant progress in recent years towards the development of new targeted therapies, Cancer remains a largely unmet medical need and the leading cause of death in industrialized countries [1]. The incidence of cancer is continuously increasing and is often associated with a variety of factors, including genetic predisposition, infectious agents, exposure to mutagens, as well as lifestyle factors [2]. Cancer is linked to the occurrence of genetic and epigenetic changes [3] and tumor cells harbor hundreds of these modifications, a fact also demonstrated through the introduction of high throughput sequencing to analyze the cancer genomes [4]. The accumulation of several genetic changes which results in the expression of several modified gene products during tumor growth implies that cancer cells can be recognized as foreign entities and potentially eliminated by our immune system, and is at the basis of the theory of immunosurveillance [5].

Notwithstanding, cancer immunotherapy has seen many more clinical failures than successes. However, very recently major breakthroughs have been achieved which have led us to believe that this approach may become an established therapeutic modality for the treatment of cancer within the next decade. As a matter of fact the importance of cancer vaccines recently increased following the demonstration that Sipuleucel-T, an immune cell-based vaccine for the treatment of hormone refractory prostate cancer, is capable of extending the overall survival of cancer patients [6], leading to FDA approval of this cancer vaccine with the name of Provenge^®^ (Dendreon) [7]. This approval has propelled renewed enthusiasm in an area that has long suffered major setbacks.

In this review we will focus mainly on the various technologies being utilized for therapeutic cancer vaccines and will provide an update of their most advanced clinical developments. Major emphasis will be given to Adenoviral vectors and DNA electroporation. For readers interested in more detailed descriptions of cancer immunology and immunotherapy we suggest the recent excellent reviews by Palucka *et al.* [8] and Klebanoff *et al.* [9].

## 2. Types of Cancer Vaccines

Different technologies are being employed for the development of cancer vaccines. In this review we will divide them into two main categories: Non Genetic Vaccines and Genetic Vaccines. The main advantage of genetic vaccines is that they allow (a) endogenous expression of the antigen of interest by muscle and/or antigen-presenting cells, which maximizes antigen processing through the endogenous pathway and epitope display on MHC class I molecules; (b) appropriate molecular engineering of the targeted tumor antigen, which helps in significantly boosting self-antigen immunogenicity and breaking tolerance.

**Figure 1.**
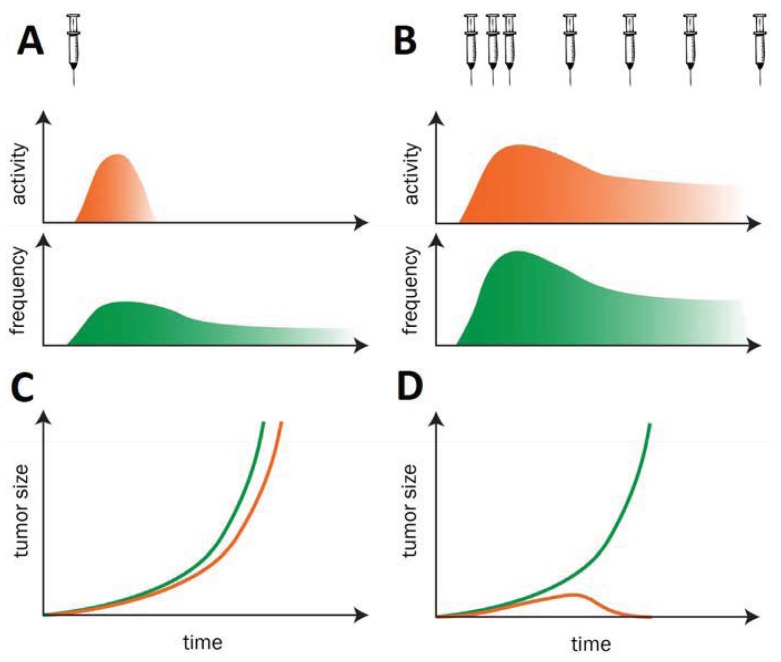
Activity of cancer vaccines: relationship between vaccine immunogenicity (activity and frequency of immune cells), repeated administration and antitumor efficacy. (**A**) Immunization with a single vaccine dose induces a limited expansion of CTL precursors (CTLp), T helper (Th) and/or B-cell producing antibodies and the activity is limited to a short period of time; (**B**) A prolonged persistence of antigen in lymphoid organs - as is reached by repetitive immunization - is necessary to increase the CTLp/Th frequency and/or antibody titer; (**C**) A single immunization does not change the tumor growth kinetic (red) in comparison to the control group (green); (**D**) Repetitive immunizations are necessary for the rejection of established tumors.

Before reviewing in detail individual technologies and their current progress we would like to stress a guiding concept for therapeutic cancer vaccine development: ideally cancer vaccines should be administered for a prolonged period of time (possibly as chronic treatment) without loss of immunogenicity. Cancer vaccines aim at breaking tolerance against self-antigens. When tolerance is broken, if vaccinations are discontinued, self-responses tend to vane due to the presence of mechanisms of tolerance. Hence, it is necessary to sustain self-immunogenicity against Tumor antigens in order to induce protective immune responses, with efficient T helper/CTL activation and long-term immunological memory. These goals are similar as in many infectious diseases and have the potential to achieve long term antitumor efficacy (Figure 1). This concept has been demonstrated in a variety of preclinical models [10] and applied in cancer vaccine human clinical trials [11].

## 3. Non Genetic Vaccines

Whole cell vaccines, perhaps the oldest approach, utilize irradiated cancer cells, either autologous or allogeneic, as source of tumor antigens either in combination with adjuvants, or genetically modified [12]. They have been shown to induce tumor-specific immunity and durable anti-tumor responses in a number of Phase I and II trials [13]. However, in the past few years several high-profile Phase III trials, including those conducted using the promising GVAX technology (see below) have failed to meet the predefined endpoints [14].

The mechanism of action of this type of vaccine is the cross-presentation of cell-derived TAAs to specific cytotoxic T lymphocytes (CTL) *in vivo*. Cross-priming is facilitated by Dendritic Cells (DCs) being activated by adjuvants or cytokines such as GM-CSF. The most advanced autologous vaccines are MVax^®^ (Avax Technologies) [15], OncoVax^®^ (Vaccinogen) [16] and Reniale^®^ (VCC Medical BV) [17], all in Phase III clinical trials for melanoma, colorectal and renal cancer respectively. In the case of Mvax^®^ autologous tumor cells are modified with DNP and re-administered to the patients. In the case of OncoVax^®^ irradiated patient-specific tumor cells are sadmixed with frozen Bacillus Calmette-Guerin (BCG) as an adjuvant.

The advantages of using allogeneic cells are obvious: (1) utilizing antigenically well-defined cell lines provides access to a sustained and virtually unlimited source of TAAs; (2) the use of cell lines allows for highly standardized large-scale production of vaccines; (3) using a single batch of allo-vaccines for all vaccinees, regardless of HLA haplotype, eliminates variability in the quality and composition of the vaccines and facilitates reliable comparative analysis of the clinical outcome; and (4) eliminating the need for the continuous production of tailor-made individual vaccines simplifies the logistics and reduces the laboriousness of the vaccine production and delivery process thus incrementing its cost-effectiveness. In the GVAX^®^ technology [18] tumor cells are transduced with GM-CSF. As mentioned above previous Phase III trials with GVAX^®^ have failed to meet their clinical endpoints [19]. However BioSante Pharmaceuticals Inc. have recently restarted development of GVAX^®^ in a Phase II trial in prostate cancer patients supported in part by the Prostate Cancer Foundation. Additional allogeneic vaccines are HyperAcute Pancreas^®^ (NewLink Genetics Corporation) and Lucanix^®^ (NovaRx Corporation). Lucanix^®^ consists of four non-small cell lung cancer cell lines that have been gene-modified to block TGFβ, a cytokine known to inhibit immune responses against cancer [20]. In HyperAcute Pancreas^®^ a mixture of irradiated allogeneic cells are genetically modified to express α(1,3)-Galactosyl residues linked to cell-surface lipids and proteins. The αGal epitopes are believed to work by exploiting the natural presence of anti-Gal antibodies to induce tumor recognition, opsonization and uptake by APCs [21]. Canvaxin (CancerVax) is another whole cell vaccination approach that entered phase III testing. It is an allogeneic melanoma vaccine (irradiated cells from 3 melanoma cell lines) given intradermally with BCG. In a nonrandomized phase II study in patients with stage IIIA/IV melanoma, median OS was 23 months compared with 7 months for historical controls. Survival has correlated with vaccine DTH responses and antibody induction in several studies [22]. However, Canvaxin did not demonstrate efficacy in a phase III study as a post-surgical adjuvant treatment for patients with advanced-stage melanoma and the company announced its discontinuation.

Immune cell-based vaccines, of which Sipuleucel-T is the most advanced are patient-specific whole cell vaccines which utilize, at least in part, professional antigen presenting cells (APCs) isolated from the patient manipulated *in vitro* both to present the tumor antigen(s) of choice and deliver features which facilitate homing to secondary lymphoid organs, thus inducing Th1 responses. APCs (e.g., DCs) can be loaded with tumor antigens in the form of peptides, proteins, tumor lysates, and mRNAs. Alternatively, they can be fused with tumor cells or infected with viral vectors encoding tumor-associated antigens [12]. The preparation of a product like Sipuleucel-T initially involves leukapheresis to obtain the peripheral blood of the patient, and this leukapheresed specimen is then transferred to the company manufacturing facility. The cell pellet containing APCs (predominantly CD54^+^), T lymphocytes (CD3^+^), B lymphocytes (CD19^+^), monocytes (CD14^+^), and natural killer cells (CD56^+^) is exposed to PA2024, an engineered antigen-cytokine fusion protein consisting of PAP (Prostate Acidic Protein) and GM-CSF. Again, GM-CSF facilitates uptake of the fusion protein by DCs and promotes DC stimulation. PAP is the tumor antigen used in this vaccine approach. The final product is transported to the patient at 4 °C and infused intravenously within 8 h of formulation. Despite the above described technical complexity, Sipuleucel-T, was recently awarded Food and Drug Administration (FDA) approval based on a successful Phase III trial showing improvement in overall survival (OS) in men with asymptomatic or minimally symptomatic metastatic advanced castrate resistant prostate cancer (CRPC).

There are several features of the clinical development of DC- and immune cell-based cancer vaccines bear several features that make this technology not ideal for large scale application. The first aspect is the difficulty in setting up standardized procedures for reliably producing functioning APCs. Currently, demonstrating that each preparation has the same levels of processed and presented antigen and activates an equivalent immune response after administration is problematic. This is true for multiple preparations given to one patient and for different preparations given to many patients (intrapatient and interpatient variability). Quality control in processing cellular products is critical to the integrity of the product. Large amounts of autologous peripheral blood mononuclear cells must be cultured in the presence of several cytokines making their off-the-shelf marketability impossible. There are critical issues not only in ensuring the proper maturation status of the cells but also in the precise selection of appropriate subsets required to elicit the desired response. Other aspects include the high manufacturing costs and the huge amount of labor required to produce a viable product within a short time frame. One can assume that these limitations may also negatively impact upon vaccine efficacy in patients. Indeed, while several studies tend to suggest that the optimal regimen for therapeutic vaccines is a chronic treatment over a prolonged period of time [23], Provenge^®^ is currently administered only as a three dose treatment [7]. Hence it is reasonable to hypothesize that the efficacy of this vaccine would be superior if it could be produced in a more straightforward manner and at lower costs, thus allowing for extended treatments.

Heat shock protein based vaccines use heat shock protein (HSP)-peptide complexes, as natural host vector for vaccination [24]. Heat shock proteins are intracellular molecules of a family characterized by members of similar molecular mass (such as hsp70 and hsp90) that act as chaperones for a repertoire of peptides, including normal self-peptides and antigenic peptides. During both protein synthesis and breakdown, heat shock protein complexes are released from cells, still associated non-covalently with peptides. Release by necrotic cells functions as an endogenous danger signal as well as a method to cross-present antigens to DCs. In fact, DCs have a specific receptor for heat shock proteins (CD91) and its engagement leads to their maturation. HSPs complexed with antigenic peptides have been shown to efficiently deliver peptides into the MHC class I processing pathway thus generating cellular immune response. The immunogenicity of tumor-derived HSP-peptide complexes, like the immunogenicity of experimentally induced tumors in mice and rats, has been shown to be individually tumor-specific and not tumor type specific. These observations have led to the conclusion that the relevant tumor-antigenic, immunoprotective peptides are derived from unique rather than shared tumor antigens.

The tumor specific HSP-complexes vaccine named HSP peptide complex-96 (HSPPC-96 or Oncophage^®^ or Prophage, previously Vitespen; Agenus, Lexington, MA, USA) has shown itself capable of inducing MHC I-restricted immune responses in a range of tumor types, and clinical responses in patients with earlier-stage disease, in line with previously published data on cancer vaccines. Vitespen is almost devoid of side effects aside from minor injection-site reactions. However, when it was tested in Phase III trials in kidney cancer it failed to meet the predefined endpoints for registration by EMA, but nevertheless it has been approved in Russia as Oncophage^®^ for the adjuvant treatment of kidney cancer patients at intermediate risk for disease recurrence. Currently Agenus is conducting trials of Oncophage for recurrent and newly diagnosed glioblastoma [25].

An additional approach is that of peptide based vaccines. This was conceived decades ago following the identification of a wealth of CD8^+^ and CD4^+^ epitopes in several tumor antigens, mainly in studies of melanoma [26,27]. Peptides can be synthesized in a standardized manner and their cost of production is relatively low, thus making peptide vaccination the technology of choice for a number of groups. Despite the convincing rationale, promising preclinical results and the frequent induction of antigen-specific immune responses, peptide-based cancer vaccines have yielded relatively poor results in the clinical setting. The results of the Phase III clinical trial in 676 metastatic melanoma patients deserve a particular mention. This compared the efficacy of a gp100 peptide vaccine, with that of the fully human anti CTLA-4 antibody ipilimumab, or with combined agents [28]. When compared to gp100 alone, ipilimumab improved median overall survival from 6.4 to 10.1 months (hazard ratio for death, 0.68; P < 0.001), but more importantly no difference in survival was detected between the Ipilimumab alone *versus* ipilimumab plus vaccine groups (median overall survival 10.1 *versus* 10.0 months, hazard ratio with ipilumumab plus gp100, 1.04; P = 0.76). Based on these results ipilimumab (but not the gp100 vaccine) was recently approved by FDA for the treatment of unresectable stage III and IV melanoma, under the name of Yervoy^®^ (Bristol-Myers Squibb).

One possible interpretation for the lack of efficacy of peptide vaccines in a patient population otherwise responsive to immunotherapy is the necessity to generate a polyclonal immune response directed simultaneously against several MHC class I epitopes. This could not be achieved in the clinical trial cited above because it made use of a single-epitope peptide. In spite of this huge limitation other single peptide vaccines are still in advanced clinical development. This is the case of Stimuvax^®^ and GV1001. Stimuvax^®^, otherwise called BLP25 liposome vaccine (Merck Serono/Oncothyreon) is a liposome-encapsulated peptide vaccine consisting of a synthetic peptide derived from the mucin 1 (MUC-1) antigen that is overexpressed on the cell surface of many epithelial tumor cells, as well as on the surface of some B-cell lymphoma and multiple myeloma cells [29]. Its most advanced development is for the treatment of NSCLC in two Phase III trials named START and INSPIRE [30]. GV1001 (KAEL-GemVax) is a peptide vaccine representing a 16-aa hTERT sequence [31].

In order to overcome the limitations of single epitope vaccines, alternative approaches are being undertaken which make use either of a combination of immunogenic peptides or of synthetic long-peptide vaccines. In both cases the presence of multiple epitopes should be able to elicit polyspecific CD8^+^ and CD4^+^ memory responses against tumor cells [32]. One example is the Eastern Cooperative Oncology Group Phase II Trial E1696 [33], where a mix of peptides containing multiple epitopes derived from three lineage-restricted antigens MART-1, gp100, and tyrosinase was administered in patients with metastatic unresectable melanoma in combination with IFNα or GM-CSF. Of note, the median overall survival of patients with vaccine immune response was significantly longer than that of patients with no immune response (21.3 *versus* 13.4 months; P = 0.046). Other recent interesting results in Phase II studies have been obtained by Immatics [34] where cancer patients are vaccinated with multiple tumor-associated peptides (TUMAPs) isolated from tumor specimens and identified by mass spectrometry [35]. The most advanced product, IMA901, a combination of several TUMAPs for the treatment of renal cell carcinoma, completed a Europe-wide multi-center Phase II clinical trial and has recently commenced a Phase III trial.

Isolated recombinant proteins have been successfully employed for antiviral vaccines. However, soluble proteins are poorly immunogenic and require appropriate adjuvants and delivery systems to induce immune responses following immunization. For optimal performance, antigen delivery vehicles should closely mimic the composition and immunological processing of actual pathogens; they should actively or passively target APCs such as DCs; protect the antigenic protein from degradation; direct the nature of the resulting immune response (*i.e.*, cellular *versus* humoral responses) and lastly, induce APC maturation by interacting with elements of the innate immune system such as Toll-like receptors (TLRs). Several strategies have been reported including directly conjugating TLR ligands to protein antigens or co-encapsulating immunostimulatory agents and proteins in liposomes or hydrophobic polymeric particles [36].

The most advanced product of this type is that being pursued for the development of MAGE-A3 antigen specific immunotherapy (ASCI). MAGE-A3 ASCI is a therapeutic cancer vaccine directed against the tumor antigen MAGE-A3, which is overexpressed in subset of patients affected by various cancers, being developed by GlaxoSmithKline (GSK) [37]. The vaccine is delivered as highly purified recombinant protein in conjunction with GSK's own proprietary adjuvant Systems. The most advanced development for the MAGE-A3 vaccine is in a Phase III trial called MAGRIT (MAGE-A3 as Adjuvant Non-Small Cell Lung Cancer Immunotherapy), which began in October 2007 and aimed at recruiting 2270 patients randomized to ASCI or placebo. The objective of the MAGRIT trial is to investigate the efficacy of MAGE-A3 ASCI in preventing cancer relapse, when administered after tumor resection, in patients with MAGE-A3 positive stages IB, II and IIIA NSCLC and is going to be the largest-ever trial in the adjuvant treatment for NSCLC.

To summarize this section, the only vaccine which gave rise to a statistically significant survival benefit in a Phase III registration trial so far utilizes an immune cell-based approach. Several other technologies are being pursued in the search for a viable alternative capable of combining improved efficacy, with more streamlined production processes and lower manufacturing costs. Ideally the goal is to obtain a non-patient-specific off-the-shelf product which can be administered as chronic treatment.

## 4. Genetic Vaccines

Genetic vaccines are highly promising tools to elicit immune responses against a wide variety of antigens, including TAAs. Two main classes of genetic cancer vaccines are being utilized: viral vaccines and DNA plasmid vaccines.

Viral infection results in the presentation of virus-specific peptides in association with both MHC class I and MHC class II on the surface of infected cells [38]. Based on this phenomenon, several strategies have been devised to use viruses as immunization vehicles to elicit antigen-specific immune responses. In these approaches, cDNAs encoding one or several antigens, which may be wild-type or genetically engineered, are inserted into the viral vector. The resulting recombinant viruses are used to infect the host so that the selected antigen(s) may be expressed and subsequently presented to the immune system [39]. For vaccination purposes, the ideal viral vector should be safe with respect to disease-causing potential, transmissibility and long-term persistence in the host. It should enable efficient presentation of expressed antigens to the immune system, while preferably exhibiting low intrinsic immunogenicity so that it can be administered repeatedly to boost relevant specific immune responses, often necessary to break immune tolerance to self-antigens.

Tumour antigen DNA sequences have been inserted into attenuated pox vectors that cannot replicate in mammalian hosts (such as modified vaccinia Ankara, fowlpox, or canarypox). Vaccinia poxvirus (VV) was demonstrated to be safe and very effective in the induction of potent cellular and humoral immune response in several tumor model systems [40]. One successful story of a vaccine based on this technology is PROSTVAC-VF^®^ (Bavarian Nordic, Kvistgård, Denmark). PROSTVAC-VF^®^ is a vaccine against PSA that includes a number of costimulatory molecules: three well-characterized costimulatory molecules were found to be synergistic when added to the poxviral system. This triad, which includes B7.1 (CD80), ICAM-1 (CD54), and LFA-3 (CD58), is designated TRICOM and has been added to both the vaccinia priming vector and the fowlpox boosting vector. With PSA as the encoded antigen, this configuration constitutes PROSTVAC-VF^®^, vaccinia-PSA-TRICOM, and fowlpox-PSA-TRICOM. A randomized, empty vector-controlled, double-blinded Phase II study was designed and powered for the short-term end point of Progression Free Survival (PFS), but failed to find an association between vaccine treatment and slowing down disease progression. However, at 3-years post study a strong association between the treatment arm and increase in overall survival was observed [41], with 30% of PROSTVAC-VF^®^ vaccinated patients still alive *versus* only 17% of controls. The estimated hazard ratio was 0.56 (95% CI, 0.37 to 0.85), and the observed difference in median survival of 8.5 months suggests significant therapeutic benefit. Based on these highly encouraging data Bavarian Nordic is planning a larger pivotal Phase III trial.

Another Pox vector based vaccine is Trovax^®^, an MVA (modified Ankara virus) vector developed by Oxford Biomedica, Oxford, UK) directed against a tumor enriched surface marker named 5T4 [42]. Clinical trials with Trovax^®^ showed good safety profile, immunologic responses and some efficacy in relation to a defined biomarker strategy but failed to meet the primary endpoint of overall survival in a Phase III registration trial for the treatment of metastatic renal cancer. Trovax^®^ is currently undergoing a Phase II clinical trial for the treatment of hormone refractory prostate cancer. Finally, TG4010, another MVA vector vaccine, is being developed by Transgene (Strasbourg, France). It incorporates two expression cassettes: the first for the MUC1 antigen, the second for the cytokine interleukin-2 as an immune stimulant. The vaccine has been tested in breast, kidney, prostate and lung cancers with encouraging results in Phase II. For Renal Cell Carcinoma (RCC), thirty-seven patients with progressive, MUC1-positive tumors received TG4010 10^8^ pfu/injection weekly for 6 weeks, then every 3 weeks until progression, when TG4010 was continued in combination with interferon-α2a and interleukin-2. Assessments included clinical response (primary endpoint), safety, time to treatment failure (TTF), OS, and immune response. No objective clinical responses occurred, but median OS was 19.3 months for all patients and 22.4 months for combination therapy recipients. MUC1-specific CD8^+^ T cell responses were associated with longer survival [43]. The efficacy and safety of TG4010 have been assessed in a randomized, controlled Phase IIb study evaluating the therapeutic vaccine TG4010 as an adjunct to standard chemotherapy in 148 patients with advanced NSCLC. The primary objective of the study, namely progression free survival at 6 months of at least 40% in the experimental arm, was met. During this Phase IIb trial, Transgene identified a subpopulation of patients who particularly benefited from the treatment with TG4010 and chemotherapy, *versus* chemotherapy alone. This sub-population consisted of patients with normal levels of activated NK cells at baseline and represented 73% of the assessable patient population (101 out of 138 patients). The Phase IIb clinical results have demonstrated an improved clinical outcome for patients in this subpopulation with a statistically significant 6 month increase in median survival (17.1 months in the experimental arm *versus* 11.3 months in the control arm). Response rate, time to progression and progression free survival data also confirmed identification of activated NK cells as an appropriate predictive biomarker associated with the positive clinical outcome for patients with NSCLC treated with TG4010 in combination with chemotherapy. A pivotal, global, controlled Phase IIb/III trial of TG4010 in patients with advanced stage (IV) NSCLC is expected to begin at the end of 2011. The trial will involve the overall recruitment of 1200 patients with MUC-1 positive NSCLC.

## 5. Adenoviral Vectors

An emerging viral system for vaccination is Adenovirus (Ad). Ad-based vectors are very efficient vehicles for gene delivery and have been extensively characterized for gene therapy purposes [44].

**Figure 2.**
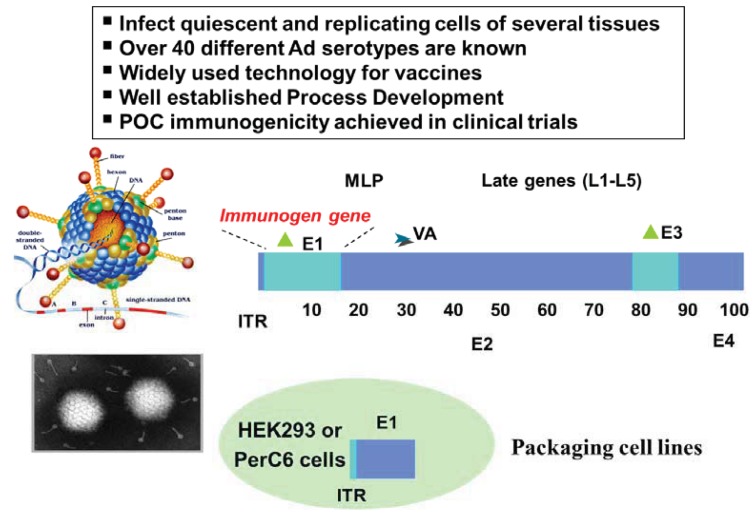
Structure and packaging of Adenoviral vectors: in the box in the upper part of the figure general features of Adenoviral Vectors are listed. In the central part is a schematic structure of the adenoviral genome. 1st generation vectors used for vaccine generation bear deletions of the E1 and E3 early genes. The Immunogen gene is inserted in place of the E1 gene. ITR = Inverted terminal repeats; MLP: Major Late Promoter; VA. Virus Associated small RNAs; E1, E2, E3, E4: early adenoviral genes. In the bottom part of the figure are schematically represented Packaging cell lines (e.g., HEK293 or PerC6 cells) which carry the complementing E1 gene integrated in a chromosomal location.

Ad vectors can be propagated to high yields in well-defined production systems that are readily amenable to pharmaceutical scale production of clinical grade compositions. These features and their well-characterized molecular genetics make recombinant Ad vectors excellent candidates for use in gene therapy and as vaccine carriers. Typically, the production of recombinant adenoviral vectors relies on the use of a packaging cell line that complements the functions of adenoviral gene products which have been either deleted or engineered to be non-functional (Figure 2).

In September 1999, the perception of using Ad vectors for gene therapy was altered when a patient affected by an inherited enzyme deficiency and exposed via the hepatic artery to a high dose of adenoviral vector succumbed to the toxicity related to vector administration [45]. Concerns were raised about continuing the use of the Ad vector system, but more importantly, there were increased efforts to fully understand its toxicity. Today it is recognized that while Ad vectors are not suitable for all applications, they offer significant advantages over other vector systems including efficient transduction of a variety of cell types, both quiescent and dividing, which make it optimal for certain applications. In particular these include protocols where the adjuvant effect of adenovirus can be beneficial to the induction of strong antigen-specific immune responses [46].

### 5.1. Adenoviruses: Biology and Serotypes

The adenoviruses comprise a large family of double-stranded DNA viruses found in amphibians, avians and mammals, which have a non-enveloped icosahedral capsid structure [47,48] and genome sizes varying between 36 and 40 kb. In contrast to retroviruses, Ads can transduce dividing and non-dividing cells, without integrating into the genome of the host cell. Ad DNA is typically very stable and remains episomal (e.g., extrachromosomal), unless transformation or tumorigenesis has occurred. Ad genes are divided into those expressed early and late during the viral lifecycle. Among the early genes, E1a and E1b proteins encode trans-activating factors that help regulate the host cell cycle. Additionally, E2 is needed for viral replication, E3 for host immune modulation, and E4 to inhibit host cell apoptosis. The Ad particle mainly contains three structural proteins: hexon, penton and fiber. The hexon protein is the most abundant of these, with each Ad containing 240 copies of the trimeric hexon molecule (accounting for 63% of the total protein mass). Viral capsids interact with the coxsackievirus adenovirus receptor (CAR) as an initial binding step, followed by secondary engagement of the integrin co-receptors α5β1, αvβ3, αvβ5, and αMβ2. Other cell surface molecules have been described for the process of Ad binding and internalization such as MHC class I [49] and heparin sulfate glycosaminoglycans [50]. When Adenovirus enters the cells, it escapes the endosome and accesses the nucleus via microtubules using nuclear pore complexes to inject its genome. Ads are naturally associated with infectious diseases such as upper respiratory infections and conjunctivitis and can cause liver, kidney, and urinary tract infections. As a consequence, Ads can successfully infect a wide variety of cells including respiratory epithelial cells, myoblasts, macrophages, hepatocytes and glial cells [51,52].

Human Ads (hAds) are divided into six subgroups (A–F) based on a number of biological, chemical, immunological and structural criteria which include hemagglutination properties of rat and rhesus monkey erythrocytes, DNA homology, restriction enzyme cleavage patterns, percentage G+C content and oncogenicity [53,54]. To date, 51 distinct serotypes have been recognized and grouped into subgroups on the basis of their hemagglutination properties and biophysical and biochemical criteria.

### 5.2. Adenoviral Vectors as Vaccine Vectors

The high immunogenicity of E1-deleted Ad recombinants has largely excluded their successful use for sustained gene therapy but this same feature has facilitated their development as vaccine carriers. Ad vaccines have been shown to induce the highest B- and CD8^+^ T-cell responses in experimental animals, including rodents, canines, and primates against a variety of immunogens derived from a number of infectious agents (e.g., viruses, parasites, or bacterial pathogens) and tumor cells, including tumor-associated antigens (TAAs) [55]. Two well-characterized human subgroup C adenovirus serotypes (*i.e.*, hAd2 and hAd5) were initially most widely used as the source of viral backbone for the majority of adenoviral vectors used for gene therapy and vaccine development. Recently, however, clinical results have shown that Ad vectors based on common human serotypes, such as serotype 5, may not be ideal as vaccine carriers. A Phase 2b trial, termed STEP with an hAd5-based vaccine expressing antigens of human immunodeficiency virus 1 (HIV-1) not only showed lack of efficacy in spite of the vaccine's immunogenicity, but also indicated an increased trend in HIV acquisition in individuals with circulating hAd5 neutralizing antibodies prior to vaccination [56]. Antibodies against the hexon protein dominate the neutralizing response elicited when humans are infected by Ad [57]. The hexon protein is composed of seven short hypervariable regions (HVRs), each of which can elicit a serotype-specific immune response [58]. Alternative serotypes from humans or nonhuman primates (NHPs), to which most of population lack pre-existing immunity against the relevant hexon protein, have been vectored and may circumvent the problems encountered with using hAd5 vectors in humans [44]. Thus, the use of uncommon Ad serotypes should bypass the issue of pre-existing immunity.

### 5.3. Alternative Ad Vectors

Vectors based on human non-subgroup C Ads have been previously reported almost exclusively for non-vaccine approaches. The Ads subgroup B (including hAd3, 7, 11 and 35) is less prevalent in the human population and the presence of anti-hAd5 antibodies does not block the transduction efficiencies of these vectors [58]. These Ads can infect cells via human CD46 (membrane complement protein) which is ubiquitously expressed in almost all human tissues, and hence they also able to infect cells lacking CAR (Coxakie Adenovirus Receptor) [59]. Sialic acid and CD80/CD86 have been shown to act as receptors for hAd subgroup D [60] and for hAd3 [61], respectively. In particular, hAd35 has been proposed as an alternative to hAd5 for vaccine delivery because of its low seroprevalence: however, comparison of hAd5- and hAd35-based vectors in mice and nonhuman primates demonstrated a lower immunological potency of the latter vector [62,63]. Similarly, two other vectors based on the rare serotypes hAd24 and hAd34 induced lower immunity than hAd5 in nonhuman primates [64].

The usage of non-human Ads as vectors to elude hAd immunity has also been proposed [65]. Several nonhuman Ads are naturally non-pathogenic to humans, have been well characterized and unlike hAds, immunity to nonhuman Ads is not expected to be prevalent in the human population. It has been shown for example that pre-existing humoral immunity in humans does not cross neutralize ovine [66], chimpanzee [67,68], canine [69,70], porcine [71] or fowl Ads [72]. This has led to the engineering of a variety of nonhuman Ad vectors which efficiently transduce several types of human and murine cells in culture being engineered [73], utilizing receptors for cell internalization and cell entry often independently from CAR [74,75]. In particular, vectors such as porcine Ad3 or bovine Ad3 are interesting because no pre-existing anti hAd5 cross-neutralizing antibodies have been detected in humans or mice [76]. Moreover, to overcome the neutralizing antibodies to hAd5, chimpanzee Ad (ChAd) vectors have been developed [77,78]. ChAd vectors such as AdC7, AdC68 and AdC1 have proven particularly effective tools as vaccines against a variety of viral antigens and diseases, such as influenza caused by H5N1 strains [79], human immunodeficiency virus type 1 in NHPs [67], rabies virus [68] and severe acute respiratory syndrome mediated by SARS-Coronavirus [80].

The strategies to avoid Ad vectors neutralization are particularly relevant to their use as cancer vaccines. In fact, one of the major obstacles to achieving a tumor specific immune response is that of overcoming central and/or peripheral T cell tolerance against TAAs and inducing CTLs that could effectively eradicate disseminated tumor metastases and subsequently maintain a long lasting immunological memory, preventing tumor recurrence [81]. Ad vectors have been proven as efficacious vaccine vectors capable of breaking immune tolerance to target antigens in transgenic mice expressing human TAAs [82,83] as well as in nonhuman primates [84]. Most importantly, Chimp Ad based vaccines such as ChAd3 have been shown to be able to induce immune responses comparable to hAd5 serotype-based vectors, break tolerance to self-tumor antigens, overcome hAd5 pre-existing immunity and achieve antitumor effects [78].

In conclusion, therefore, Ad vectors constitute a vast repertoire of agents suitable for the development of therapeutic cancer vaccines. The availability of multiple serotypes and of non-human Ad vectors should successfully overcome issues previously observed with hAd5 based vaccines.

### 5.4. Clinical Progression of Ad Vectors in Oncology

Several clinical studies using Ad vectors have been conducted in oncology. Among the others, intratumor (IT) injections of Ad containing the wild-type p53 tumor suppressor gene showed clinical efficacy when combined with chemotherapy and led to the clinical development of Advexin^®^ and Gendicine^®^. Advexin^®^ and Gendicine^®^ are recombinant Ad5 with the E1 region replaced by a human wild type p53 expression cassette containing a Cytomegalovirus (CMV) or Rous sarcoma virus (RSV) promoter, respectively [85]. In October 2003, the Chinese State Food and Drug Administration (CSFDA) approved Gendicine^®^ as the first commercialized gene therapy product in the world. Another advanced application is represented by oncolytic Ads [86]. The best known example is Onyx-015, the first engineered replication-selective virus to be used in humans. It is an Ad2/Ad5 hybrid with deletions in the E1B and E3B region and replicates exclusively in cells with inactive p53, activated p14ARF and late viral mRNA transport. Onyx-015 has been tested in more than 15 clinical trials by direct IT injection (up to 5 × 10^9^ vp) and resulted in limited antitumoral effects (objective response rate 14%) and systemic spread of the virus was not detected [87]. One of the possible reasons for these poor results is the extent to which viruses infect targeted cells. Many tumors down-regulate CAR, rendering them less susceptible to infection [88]. In November 2005, CSFDA approved H101, an oncolytic adenovirus similar to Onyx-015 (E1B-55K/E3B-deleted), for use in combination with chemotherapy for the treatment of late-stage refractory nasopharyngeal cancer [89]. It is also important to mention that oncolytic vectors others than Ad based ones are emerging as a fashionable approach. An oncolytic virus derived from HSV (Herpes simplex virus), is being actively pursued by Amgen/Biovex. OncoVEX^GM-CSF^, which selectively infects and generates GM-CSF in tumor cells resulting in increased tumor antigen presentation by recruited DCs. This agent is presently in Phase III trials of melanoma and head& neck cancer, based on highly promising Phase II trial results [90].

In specific relation to the development of cancer vaccines, several groups are currently assessing the immunologic and clinical activity of Ad vectors expressing a variety of TAAs, including prostate serum antigen (PSA), HER-2/*neu*, carcinoembryonic antigen (CEA) and telomerase (hTERT) [91]. It will be of great interest to verify how local and systemic suppression exerted by the tumor itself as well as pre-existing immunity to Ad will impact the outcome of these studies.

## 6. DNA Vaccines

**Figure 3.**
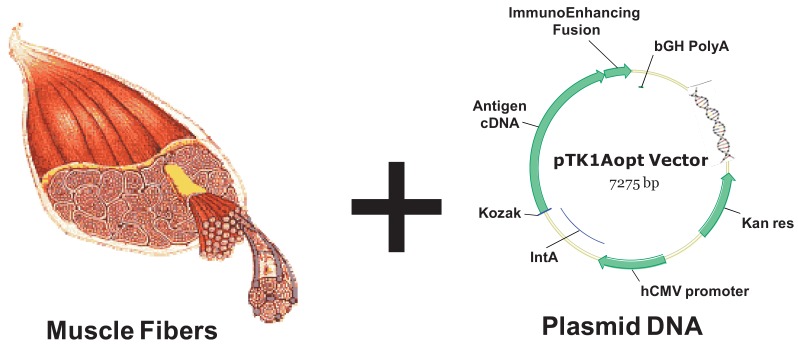
Intramuscular delivery of a DNA vaccine: in the right panel of the figure is a schematic representation of a standard expression vector used for DNA vaccines bearing an expression cassette driven by the Cytomegalovirus Intron A (CMV-IntA) promoter. The antigen cDNA can be engineered and fused to an immunoenhancing moiety. Muscle cells (left) are the major target for expression of the antigen of choice.

Inoculating plasmid DNA encoding for a protein antigen by means of a simple intramuscular or intradermal injection currently offers a vaccine approach that is easily performed, safe for host and relatively inexpensive [92]. DNA delivery vehicles consist of a gene regulated by a promoter usually showing constitutive activity (for example the cytomegalovirus early enhancer-promoter, see Figure 3). Simple injection of naked DNA sequences results in gene expression and the potential generation of immune responses. A possible mechanism of how DNA immunization works is the following: the protein antigen is produced by target cells (usually myocytes or fibroblasts, depending on the injection route) and the secreted antigen may be taken up by host APCs, processed, and cross-presented to the immune system in the draining lymph nodes. Direct transfection of rare APCs residing at the injection site has also been demonstrated. However, target cells lack the co-stimulatory molecules needed as part of the CTL activation process, therefore DNA vaccination is in general poorly efficient unless an inflammatory stimulus is applied in parallel.

The ability to make new DNA vaccines without needing to handle a virulent pathogen or adapt it for manufacturing purposes opened the suitability of this vaccine technology not only for development of cancer vaccines but also for emerging and epidemic pathogens. Four animal health plasmid DNA products, including two prophylactic vaccines against infectious diseases, one for the immunotherapy of cancer (Oncept™ see below), and one gene therapy delivery of a hormone for a food animal, have recently been approved by drug administration authorities. This confirms the efficacy of DNA vaccines in multiple species including also large animals such as dogs, horses and pigs. A particular mention deserves Oncept™, currently the first and only USDA-Approved therapeutic vaccine for the treatment of cancer. Oncept™ has been developed by Merial [93] through a partnership with the Memorial Sloan-Kettering Center and the Animal Medical Center in New York. It is a DNA vaccine expressing human tyrosinase (therefore defined as xenogeneic vaccine) against canine malignant melanoma [94], and delivered via a Transdermal Device, developed in conjunction with Bioject Inc [95], which delivers the vaccine without the use of a needle. Oncept™ has been shown to extend survival of dogs with stage II or III oral canine melanoma.

In mouse models, DNA vaccines have been successfully directed against a wide variety of tumors, almost exclusively by driving strong cellular immune responses in an antigen-specific fashion. However, there is still a need to improve the delivery of DNA vaccines and to increase the immunogenicity of antigens expressed from the plasmids. Clinical trials for DNA vaccines have shown that immune responses can be generated in humans, but they also highlight that increased potency is required if this vaccine technology is to be effective. For example, on the basis of the successful use of gp100, MART-1/Melan-A, and tyrosinase in melanoma preclinical models, several clinical trials were conducted or are still ongoing. In an initial study, patients with metastatic melanoma were immunized with human gp100 (hgp100) expressing naked plasmids showing no clinical or immunological responses and indicating that the delivery system and adequate costimulation play an important role for the success of this approach [96]. Xenogeneic DNA vaccines have also been evaluated in two recent phase I trials using mouse gp100 (mgp100) DNA vaccines alone or in combination with the human homologue. Melanoma patients immunized with the xenogeneic vaccines developed hgp100-specific and IFN-γ-secreting CD8^+^ T cells, and 30% of them showed an immune response [97,98]. Plasmid DNA encoding for PSMA were used in two phase I/II studies to immunize patients with prostate cancer. Costimulation of plasmid DNA with the molecule CD86 led to delayed-type hypersensibility to PSMA in half of the patients, but additional boosting with an Ad was necessary to induce immunity in all of them. However, the success of these trials is difficult to interpret due to the heterogeneity of the patient populations [99,100]. Phase I/II trials of idiotypic vaccination for follicular B-cell lymphoma using a genetic approach were also conducted [101]. Vaccines encoding individual DNA idiotypic single-chain variable fragment (scFv) fused to Tetanus Toxoid Fragment C (TTFrC) were delivered as naked DNA by i.m. injection in patients with follicular lymphoma in clinical remission following chemotherapy, and were able to develop cellular or/and humoral antiidiotype immune responses in 38% of patients over a period of several months [102].

Although the reasons why DNA vaccines fail to induce potent and effective immune responses in humans have not yet been elucidated, it is reasonable to assume that low levels of antigen production, inefficient cellular delivery of DNA plasmids and, most importantly, insufficient stimulation of the innate immune system have roles in the low potency of DNA vaccines [103]. Therefore, further optimization of DNA vaccine strategies is necessary. In order to design better DNA vaccines, regimens, plasmid dose, timing of doses, appropriate adjuvants, delivery systems and/or routes of vaccination must be taken into consideration.

Generally, DNA plasmids are delivered using a number of physical approaches including tattooing, Gene gun, Ultrasound, Laser and DNA electroporation [104]. Here we will particularly focus our attention on DNA electroporation.

### 6.1. DNA Electroporation

*In vivo* electroporation of plasmid DNA (DNA-EP) has emerged as a safe method resulting in greater DNA uptake leading to enhanced protein expression in the treated muscle, and in a concomitant increase in immune responses to the target antigen in a variety of species [105-107]. Because of its properties, DNA-EP is a desirable vaccine technology for cancer vaccines as it is repeatable several times, as required for the maintenance of anti-tumor immunity [23,108]. This approach uses brief electrical pulses that create transient “pores” in the cell membrane, thus allowing large molecules such as DNA or RNA to enter the cell cytoplasm. Immediately following cessation of the electrical field, these pores seal and the molecules are trapped in the cytoplasm without causing cell death [109]. Typically, milli- and microsecond pulses have been used for EP. In addition to the increased permeability of target cells, EP may also enhance immune responses through increased protein expression, secretion of inflammatory chemokines and cytokines, and recruitment of APCs at the EP site. As a result, both antigen-specific humoral and cellular immune responses from EP mediated delivery of plasmid DNA are higher than levels achieved by intramuscular injection of DNA alone. Indeed, the addition of *in vivo* EP has been associated with a consistent enhancement of cell-mediated and humoral immune responses in small and large animals [110], supporting its use in humans.

Several devices are available for *in vivo* DNA-EP. The most advanced technologies are those being developed by Inovio Pharmaceuticals [111] and by Ichor Medical Systems [112], respectively. Both companies have set up in the past few years, also in part through partnership with other companies, articulated pipelines of DNA-EP based infectious disease and cancer vaccines. Altogether, the electroporation of DNA has been investigated in several clinical trials for cancer therapy. They include: (1) Intra-tumoral IL-12 DNA plasmid (pDNA) [ID: NCT00323206, phase I clinical trials in patients with malignant melanoma]; (2) Intra-tumoral VCL-IM01 (encoding IL-2) [ID: NCT00223899; Phase I clinical trials in patients with metastatic melanoma]; (3) Xenogeneic tyrosinase DNA vaccine [ID: NCT00471133, Phase I clinical trials in patients with melanoma]; (4) VGX-3100™ [ID: NCT00685412, Phase I clinical trials for HPV infections]; and (5) IM injection of prostate-specific membrane antigen (PSMA)/pDOM fusion gene [ID: UK-112, Phase I/II clinical trials for prostate cancer] [113].

The most advanced DNA-EP cancer vaccine product in clinical development is VGX-3100™ currently in Phase II development for Cervical Displasia/Cancer by Inovio. This vaccine includes plasmids encoding E6 and E7 proteins of HPV types 16 and 18. Intramuscular injection of the plasmid DNA vaccine is followed by electroporation using the CELLECTRA^®^ delivering device and a Phase I dose escalation study achieved best-in-class immune responses. In that study, which treated subjects with a history of surgically treated CIN2/3, 13 out of 18 vaccinated subjects (72%) developed significant T-cell responses, with positive responses ranging from under 100 to over 5,000 spot forming units (SFU) per million cells. In the third and highest dose group, 83% (5 out of 6) had strong T-cell responses. In addition, 15 of the 18 vaccinated subjects (83%) developed antibody responses to at least one antigen with most subjects developing responses to two or more antigens. No DNA vaccine has previously achieved this rate of response. The therapy was tolerable with no significant adverse events. Other Cancer Vaccine candidates being pursued by Inovio are: (a) V930, a DNA vaccine being developed in partnership with Merck, which is designed to target cancers expressing the antigens HER-2/*neu* and/or CEA (ID: NCT00250419); (b) V934, a DNA plasmid, also being developed in partnership with Merck that encodes human Telomerase (hTERT) (ID: NCT00753415). Both vaccines are being delivered in Phase I studies using MedPulser® DNA Delivery System in combination with Ad vectors; (c) a leukemia vaccine targeting WT-1 delivered using Inovio's ELGEN-1000 automated device.

### 6.2. Additional Approaches to Improve DNA Vaccine Immunogenicity

DNA vaccines offer great promises, also because they present ample margins for further improvements in efficacy. Hence, several attempts are being made to enhance immunogenicity of DNA vaccines. Interestingly in most cases these enhancements are incremental with the use of DNA-EP.

The most promising avenues are (a) use of codon-optimization; (b) generation of fusion products with immunoenhancing proteins; (c) co-delivery of genes, RNAi molecules or TLR agonists affecting immunoregulatory signaling pathways. The first approach aims at enhancing the expression of the encoded antigen in eukaryotic cells transduced *in vivo*. It is well known that elevated percentages of AU in eukaryotic mRNAs result in instability, increased turnover, and low expression level of the encoded protein(s) [114]. Furthermore, selection plays a major role in the determination of codon usage in all organisms studied so far. As a matter of fact, in highly expressed genes a narrow set of codons is used and these codons correspond to the more abundant tRNA species [115]. This minimizes the risk of tRNA depletion during translation. In contrast, some codons for rare tRNAs in a gene may be true bottlenecks. In such cases, it has been shown that substitution of these rare codons may increase the yield dramatically [116]. Very simple algorithms have been created therefore for the design of codon optimized cDNAs [117]. We have shown that optimization of the codon usage of several TAA genes in a variety of experimental systems leads to significant enhancement of expression and increased immunogenicity [83,84,106,107,118]. The second approach consists in the creation of DNA vaccines containing tumor antigen sequences fused to other genes. They are often but not exclusively microbial genes. The fused microbial protein engages local CD4^+^ T cells to provide help for anti-tumor immunity, and to induce more potent CD8^+^ T cell responses able to suppress tumor growth [82]. Among the most studied fusion DNA vaccines are those that incorporate tumor-derived sequences fused to an engineered domain of tetanus toxin [119] or to the B subunit of the heat-labile enterotoxin from *E.coli* [120]. The third and final approach is directed to simultaneously potentiate the activation pathways responsible for the induction of immune responses against tumor antigens. We have shown for example that this is possible by delivering TLR agonists [23,121,122], or also through the use of interfering RNAs targeting apoptotic genes [123]. In this last case, we believe that the enhancement of immune responses against the target tumor antigen, and the enhanced protection from tumor development are consequent to inhibition of cell death of transduced cells and sustained tumor antigen expression.

In conclusion, DNA vaccines have several potential advantages over peptide and recombinant viral vaccines. They are simpler and cheaper to produce and DNA immunization is not associated with the anamnestic immune response against the vector, which is instead responsible for the inactivation of viral vectors after repeated injections. On the other hand, their efficacy requires improvement, especially for cancer vaccines targeting ‘self’ antigens where immunological tolerance may block their function.

## 7. Heterologous Prime/Boost

There are emerging evidences that vaccination schedules comprising more than one delivery method against the same antigen(s) (*i.e.*, genetic vectors, genetic vector/protein, genetic vector/peptides, *etc.*) are best positioned to overcome the ‘therapeutic immunity’ threshold and adequately harness the immune system to fight cancer [124]. For example, the sequential administration of plasmid DNA and an adenoviral vector in different combinations may result in synergistic immune activation. Indeed we have shown in preclinical murine and primate models that this heterologous prime-boost regimen induces 10- to 100-fold higher frequencies of T cells than do naked DNA or recombinant viral vectors alone [83,84,120,125]. A further advantage of heterologous prime/boost protocols comprising the sequential use of Ad and plasmid DNA is that one can exploit the strong immunogenicity of Ad as best priming agent to break tolerance, while DNA can be used for repeated boosting because of the lack of anamnestic responses against the vector.

We have recently demonstrated that this Ad prime/DNA boost vaccination approach when directed against dog telomerase is able to give rise to significant increase in survival in dogs affected by malignant lymphoma [108]. Another approach, utilized in the development of PROSTVAC-VF^®^ is the sequential administration of two different viral vectors carrying the same tumour antigen gene, which also bypasses the limitation of the development of neutralizing antibodies to the viral backbone by boosting with a different vector without shared viral epitopes [41].

## 8. Toxicology of Cancer Vaccines

Cancer Immunotherapy has been initially advocated as being very specific for cancer cells and to have fewer side effects than conventional therapies. This concept is confirmed by reports from cancer vaccines clinical trials of cases of patients experiencing complete responses in the absence of any serious adverse event [126]. An even more significant example is the very benign toxicity profile of Sipuleucel-T [127].

It has to be pointed out however, when examining large trials, that vaccine-related adverse events, albeit rare and usually mild, are being observed. For example in a recent meta-analysis of 500 cases of advanced cancer patients treated with therapeutic peptide vaccines, 6 severe adverse events (SAEs) were related to the vaccine itself [128]. They consisted mainly in local skin reactions or cellulitis around the injection sites. In some cases, more systemic effects such as edemas of the head and neck regions, colitis, rectal bleeding and bladder-vaginal fistulae were reported.

The occurrence of autoimmunity is particularly evident in the case of therapies with systemic immunomodulators more than with cancer vaccines. Indeed, Immune-related adverse events (IRAEs) are being commonly observed in patients after CTLA-4 blockade and most likely reflect the drug mechanism of action and corresponding effects on the immune system [129]. Immunotoxicities resulting from Ipilimumab treatment can range from relatively minor conditions, such as skin depigmentation, to severe toxicities against crucial organ systems, such as liver, heart and lung. In the Ipilimumab registration trial Grade 3 or 4 IRAEs occurred in 10 to 15% of patients treated and seven deaths were associated with IRAEs [28]. Treatment-related toxicity correlates with better responses in some cases, and it is likely that serious adverse events from immune-mediated reactions will increase in frequency and severity as immunotherapeutic approaches become more effective [130]. Hence, scientists and physicians should be on guard for SAEs associated with augmented immune responses and strategies will have to be developed to avoid or circumvent these side effects.

The use of viral vectors in past gene-therapy trials has been shown to cause the occurrence of leukemogenesis [131]. This phenomenon has been associated to the use of retroviral vectors and is due to their integration into the host genome and the activation of adjacent proto-oncogenes. It is, therefore, important to carefully analyze whether genetic vaccines that make use of either naked DNA or viral vectors may raise similar issues. It has to be pointed out, however, that genetic cancer vaccine vectors under development bear two significant differences when compared to gene therapy with retroviral vectors. First, electroporated DNA [132], Ad [133] or Pox vectors used for cancer vaccines have a very low or null chromosomal integration, respectively, in the host genome. Second, vaccines are inoculated at peripheral sites of the body such as dermal tissue or skeletal muscles which are mainly composed of terminally differentiated and mitotic quiescent cells. At any rate, Regulatory Agencies require the inclusion of genome integration and genotox studies for any new genetic vaccine as part of the documentation to be included for IND filings.

## 9. Conclusions

Significant progress in the knowledge of the immune system and in the development of innovative vaccination technologies lead us to believe that cancer vaccines have a bright future and that within the next ten years they will become an established therapeutic modality for cancer. The practical demonstration is the recent licensure of Provenge^®^ for the therapy of prostate cancer in men, and that of Oncept™ for the therapy of oral melanoma in dogs. This success will strictly depend upon the respect of the four major principles listed below:

1. Use of a well established vaccination technology capable of inducing strong multi-epitope antigen-specific T and B cell responses, while using reproducible and easily scalable technologies. In this respect we believe that the most promising technologies are based on the use of genetic vectors, such as Ad and electroporated DNA, primarily when used in heterologous prime-boost combinations;
2. Appropriate combinations of vaccines with chemotherapy and/or with immunomodulators;
3. Development of an articulated biomarker strategy in parallel with clinical development, to standardize the quantification of antigen-specific T cell responses as a pharmacodynamic measure of vaccine immunogenicity, and to pre-select the best responders to treatment;
4. A development paradigm that takes into account the evolving scenario and draws inspiration from the consistent improvement of endpoints for cancer immunotherapy trials.
